# Early postoperative interventions in the prevention and management of thyroidectomy scars

**DOI:** 10.3389/fphys.2024.1341287

**Published:** 2024-03-06

**Authors:** Nan Hong, Bin Sheng, Pan Yu

**Affiliations:** ^1^ Department of Burn and Plastic Surgery, Jinling Hospital, Affiliated Hospital of Medical School, Nanjing University, Nanjing, China; ^2^ Department of Dermatology, Jinling Hospital, Affiliated Hospital of Medical School, Nanjing University, Nanjing, China; ^3^ Department of Medical Cosmetology, Sir Run Run Hospital, Nanjing Medical University, Nanjing, China

**Keywords:** thyroidectomy, linear scars, postoperative interventions, early-stage treatment, hypertrophic scars

## Abstract

Thyroidectomy scars, located on the exposed site, can cause distress in patients. Owing to the cosmetic importance of thyroidectomy scars, many studies have been conducted on its prevention and treatment. Scar formation factors mainly include inflammatory cell infiltration, angiogenesis, fibroblast proliferation, secretion of cytokines such as transforming growth factor (TGF)-β1, and mechanical tension on the wound edges. Anti-scar methods including topical anti-scar agents, skin tension-bearing devices, and local injections of botulinum toxin, as well as lasers and phototherapies, that target these scar formation factors have been developed. However, current studies remain fragmented, and there is a lack of a comprehensive evaluation of the impacts of these anti-scar methods on treating thyroidectomy scars. Early intervention is a crucial but often neglected key to control hyperplastic thyroidectomy scars. Therefore, we review the currently adopted early postoperative strategies for thyroidectomy scar reduction, aiming to illustrate the mechanism of these anti-scar methods and provide flexible and comprehensive treatment selections for clinical physicians to deal with thyroidectomy scars.

## Introduction

Thyroidectomy is often conducted on the anterior neck, which is a highly sensitive and visible anatomic location. Thus, postoperative scarring after thyroidectomy can make patients feel distressed (Bayat et al., 2003). The cosmetic outcome of the scar after thyroidectomy has raised wide interest in thyroid surgeons (Consorti et al., 2013), and many attempts to minimize thyroidectomy scars have been performed. The thyroidectomy incision length has been shortened from a 10-cm-long Kocher’s incision to a 15-mm-long access achieved by video-assisted thyroidectomy (Chung et al., 2021). In addition, thyroidectomy conducted by robotic surgery via an intraoral approach or endoscopic surgery via a postauricular approach leaves no visible scar on the neck (Teoh et al., 2019). However, robotic thyroidectomy and endoscopic thyroidectomy are conducted by few top facilities, and the traditional 10-cm-long Kocher’s incision remained the most adopted surgical approach. Therefore, early-stage interventions to improve the cosmetic outcome of thyroidectomy incision are of critical importance. Several surgical and nonsurgical approaches have been proposed for the prevention and treatment of thyroidectomy scars (Jung et al., 2011; Ha et al., 2014; Shin et al., 2014; An et al., 2019), and these interventions have displayed varying degrees of success.

Certain body sites, such as the neck area, shoulder area, anterior chest, lower abdomen, and earlobe, which have an overly bony prominence or bear greater skin tension, are more prone to an exaggerated scarring response (Aarabi et al., 2007; Ogawa, 2008). Patients of Afro-Caribbean descent and those with a personal or family history of hypertrophic scars or keloids are more likely to suffer from an exaggerated scarring response. Young age (less than 30 years) is reported to be another risk factor, especially for keloids (Berman et al., 2007; Profyris et al., 2012). However, little can be done to change the innate tendency of certain individuals or body sites, and further research aiming to reduce this scar formation risk is necessary.

Recent fundamental research also identified the major factors considered to affect scar formation and prevention, which are inflammatory cell infiltration, angiogenesis, fibroblast proliferation, secretion of cytokines such as transforming growth factor (TGF)-β1, and mechanical tension on wound edges (Bayat et al., 2003; Korntner et al., 2019; Zhang et al., 2020). Many anti-scar methods have emerged that target these scar formation factors, including topical drugs, tension-bearing devices, local injections, and lasers. However, so far, studies on anti-scar interventions concerning thyroidectomy scars remain fragmented. Therefore, in this review, we illustrate the mechanism and evaluate the clinical impacts of the current anti-scar methods for thyroidectomy scars.

## Topical anti-scarring drugs

Topical anti-scar ingredient, mainly including silicone, asiaticoside, and onion extracts, lends itself as the most common and convenient postoperative scar prevention method. Silicone products could increase hydration in scars and local skin temperature under the occlusive membrane, leading to a decrease in scar size (Chang et al., 1995; Borgognoni, 2002; Chan et al., 2005; Berman et al., 2007). Silicone gel sheeting is a self-adhesive and semi-occlusive dressing for anti-scar purposes. Silicone gel sheeting is beneficial for creating a closed-wound environment, which will increase the hydration of the cuticle, contributing to the stability of mast cells, and inhibit the release of inflammatory cytokines (Trace et al., 2016). In addition, the hydrated occlusive wound environment, which was created by the silicone gel cream, can reduce capillary permeability and subsequently reduce the release of regeneration cytokines (Quinn et al., 1985; Sawada and Sone, 1990). The use of silicone gel sheeting alleviates symptoms like pain and itching associated with scarring (Trace et al., 2016). Previous studies proved silicone gel sheets or silicone oil-based cream to be effective in limiting hypertrophic growth of postoperative scars (Juckett and Hartman-Adams, 2009). In addition, small molecule types of silicone in silicone oil cream can penetrate the skin and inhibit the proliferation of fibroblasts, resulting in reduced collagen deposition (Kuhn et al., 2001). A randomized controlled trial of patients undergoing skin surgery showed that patients who used silicone cream after removal had a significantly reduced formation of hyperplastic scars and keloids (de Giorgi et al., 2009).

It is suggested that silicone gel sheets should be applied as early as 2 weeks post-operation (Son and Harijan, 2014), especially for patients with predisposing factors for hypertrophic scars. The gel sheet should be trimmed slightly larger than the scar and applied continually for up to 6 months after the operation. Meanwhile, the silicone oil-based cream could be used alternatively in locations where sheet attachment is difficult (Sawada and Sone, 1990). The silicone cream is recommended to be topically applied 3–4 times a day and massaged for 5–10 min with each application (Son and Harijan, 2014).

Asiaticoside is a white needle-like crystal extracted from the traditional herbal medicinal plant, *Centella asiatica*, a plant of the umbelliferone family, which has been used for many years for the treatment of dermal disorders, such as venous insufficiency and microangiopathy (Shukla et al., 1999). Asiaticoside can promote collagen crosslinking and re-epithelialization, leading to faster wound maturation and contraction, thus reducing erythema and pigmentation of hypertrophic scars. An *in vitro* experiment also demonstrated that the addition of asiaticoside can inhibit the proliferation of fibroblasts and downregulate the expression of TGF-β, leading to the prevention of excessive scarring (Cheng et al., 2004; Ju-Lin et al., 2009; Zoumalan, 2018).

Onion extract gel, with a much lower price, was proved by a recent clinical trial to have compliance, side effects, and efficacy similar to those of silicone gel in making surgical scars less distinct (Song et al., 2018). Onion extract possesses anti-inflammatory, antibiotic, and collagen-degradation properties (Augusti, 1996), which is suitable for scar management. A recent clinical trial proved that onion extract gel can help restore the stratum corneum barrier, reduce transdermal dehydration, and inhibit the keratinocytes related to scar formation. Topical onion extract combined with silicone derivate gel is proven to achieve safe and effective results in the prevention of hypertrophic surgical scarring (Jenwitheesuk et al., 2012). However, the anti-scar effect of onion extract is still controversial, and many researchers consider onion extract to have a limited effect in relieving redness and itching symptoms associated with surgical scarring. With few existing studies, most scholars agreed that the therapeutic mechanisms of onion extract gel on hypertrophic scars mainly depend on reducing inflammation and fibrotic cell proliferation, inhibition of connective tissue components (proteoglycans and collagen), moisturizing scar tissue, and antimicrobial capacity (Willital and Heine, 1994; Maragakis et al., 1995; Jackson and Shelton, 1999; Hosnuter et al., 2007).

Other related anti-scar drugs are also listed in few studies, such as hyaluronic acid, which supplements the extracellular matrix (ECM) and prevents scar formation (Bullard et al., 2003). Another anti-scar component worth mentioning here is curcumin, which regulates the inflammatory response during wound healing by inhibiting the production of IL-1 and TNF-α that activate monocytes and macrophages. Curcumin can also accelerate wound re-epithelialization, therefore yielding a better scar outcome (Tejada et al., 2016). A 16-week-long comparative study indicated that vitamin E lotion could achieve significant improvements in volume, length, induration, erythema, and pigmentation alteration of hypertrophic scars (Perez et al., 2010), while topical calcipotriol cream showed no statistically significant improvement in the prevention of hypertrophic scars (van der Veer et al., 2009).

## Skin tension-reduction methods

The linear incisional scar response, which is often generated by thyroidectomy, is determined by modifiable factors including incision design, aseptic techniques, complete hemostasis, atraumatic handling of soft tissue, and skin tension (Larson et al., 2010). High skin tension is well known to be a critical causative factor for the development of wide and hypertrophic scars in humans (Wong et al., 2011; Suarez et al., 2013; Suarez et al., 2014). A cutaneous tension-reducing approach is mandatory in early-stage postoperative scar prevention and management. During surgeries, clinicians strive to make skin incisions that follow the relaxed tension lines on the body, the so-called Langer lines (Wilhelmi et al., 1999), aiming to minimalize incision tension.

In the case of sutured wounds, the epidermis can regenerate within 7–10 days, allowing both the patient and the doctor to believe that the wound has fully healed. In fact, it takes up to 3 months for the dermis to return to 90% of its normal strength. This long-term vulnerability to mechanical forces triggered by inflammation means that immature scars must be provided with long-term external mechanical support until maturity, which indicates the necessity of skin tension-reduction devices (Akaishi et al., 2008; Ogawa et al., 2011; Ogawa et al., 2016; Ogawa et al., 2021).

The skin tension-bearing tape or device is designed to shield the healing incision from the natural tension that is inherent in any break in skin that must be pulled together to close a wound (Atkinson et al., 2005; Longaker et al., 2014). It is estimated that tensile strength across an incision is only 3% of that of uninterrupted skin at 1 week after the surgery and increases to 20% by the third week when scar remodeling begins and to 80% after 12 weeks. The skin load-bearing tape or device is supposed to be applied across the incision for at least 12 weeks to reduce the tension through the whole wound remodeling phase. In addition, the load-bearing tape or device is suggested to be applied on the incision of convex skin surfaces rather than that of flexor crease locations (Atkinson et al., 2005; Son and Harijan, 2014).

The simple total surgical excision of keloids and hypertrophic scars with high recurrence rates associated is often disappointing for patients with cutaneous scars. Novel flap surgery and skin graft methods have been developed to release wound tension and change the orientation of the scar. The subtotal excision with a rim of keloids left behind was reported to achieve a better outcome owing to low wound tension and decreased collagen synthesis (Engrav et al., 1988). “Z” and “W” plastic surgery combined with postoperative nonsurgical adjuvant therapy are classic flap surgeries for successful management of small hypertrophic scars (English and Shenefelt, 1999). However, the evidence base of surgical strategies for scar treatment is, overall, inadequate and should be cautiously adopted in clinical practice.

Cryotherapy is another method for volume and tension reduction of hypertrophic scar tissues. A cryotherapy agent (most commonly liquid nitrogen) can induce scar tissue destruction by direct cell-freezing effects and cause vascular stasis during the thawing phase (Zouboulis, 1998). It is reported that the use of the intralesional cryo-needle, which is superior to the conventional open-spray method, can achieve approximately 60% volume reduction in hypertrophic scars and keloids with fewer adverse reactions after a single intralesional cryogenic treatment (Har-Shai et al., 2003; Har-Shai et al., 2006; Har-Shai et al., 2008).

## Local injections of botulinum toxin, steroids, and chemotherapy agents

Botulinum toxin type A (BTA) is a potent neurotoxin that indirectly blocks neuromuscular transmission with inhibition of exocytosis of acetylcholine, leading to functional denervation of striated muscles and glands for 3–6 months (Noland et al., 2016; Sundaram et al., 2016). A major factor determining the final cosmetic appearance of a cutaneous scar is the tension acting on wound edges during the healing phase (Huang et al., 2013). Dermal injections of botulinum toxin type A and their diffusion into the surrounding muscles could reduce the mechanical tension on the wound edges (Hsu et al., 2004; Zhibo and Miaobo, 2008; Kim et al., 2014). A randomized, double-blind, placebo-controlled primate study indicated that BTA injections would lead to scar improvement by causing paralysis of surrounding muscles, thereby reducing continual tension on the wound (Larrabee, 2000). Another clinical retrospective analysis based on the medical records of 96 thyroidectomy patients also demonstrated that BTA injections act on the muscles surrounding the scar to reduce the pathologic role of mechanical stress (Kim J. H. et al., 2012).

*In vitro* and animal experiments have demonstrated that the mechanism of scar management by botulinum toxin injections involves suppressing infiltration by inflammatory cells and delaying the fibroblast cell cycle (Lee et al., 2009; Prodromidou et al., 2015). TGF-β-secreting macrophages are the most important inflammatory cells during the acute wound healing phase (Mahdavian Delavary et al., 2011). However, studies proved that BTA plays an important role in the inhibition of capsule formation through the TGF-β/Smad signaling pathway (Kim et al., 2016). In addition, BTA decreases the expression of connective tissue growth factors, which is a downstream regulator of TGF-β1 secreted by macrophages, thereby suppressing scar formation by fibroblasts (Xiao et al., 2011). However, it remains controversial whether BTA increases or decreases the vascular endothelial growth factor (VEGF) (Kim et al., 2009; Arnold et al., 2014). A few studies have shown changes in the melanin index after BTA injection, but there was no significant difference in melanin index between patients injected with BTA and the control groups in both studies (Zhu et al., 2016; Zhu et al., 2017). The presence of botulinum toxin type A reduces inflammation in the wound healing process, which leads to a significant difference in scar erythema, as reported by clinical research (Arnold et al., 2014).

Therefore, botulinum toxin type A has been applied clinically to prevent postoperative linear scars at multiple sites, including thyroidectomy scars (Kim et al., 2014; An et al., 2019), cleft lip scars (Chang et al., 2014), and maxillofacial and neck scars (Zhang et al., 2016). Three types of BTA-delivering methods have been reported: 1) for thyroidectomy scars: BTA injections were administered into the dermal layer 0.5 cm from the incision line in 2 rows (cephalad and caudad to the incision), 5 U at a time, at 1.5-cm intervals, and the total dose never exceeded 60 U (Kim et al., 2014); 2) for small-scale linear scars, injections were administered into the dermis 0.2 cm away from the wound edge with 5 U each site (Chang et al., 2014); 3) for normal-scale linear scars: BTA injections were administered into the dermal layer 0.5 cm away from the incision scar with 10 U each site at 1-cm intervals (Zhang et al., 2016). In addition, no obvious side effects of BTA treatment of linear scars have been reported by previous literature studies. The dose and site of BTA injections could be reduced according to the scale and the treatment response of the scar.

The time of injection in these studies varied from the day of surgery to within 3 days, 5 days, and 10 days after surgery (Ziade et al., 2013; Kim et al., 2014; Zhang et al., 2016; Hu et al., 2018; Lee et al., 2018). A clinical research based on 30 adult patients indicated that the operation-day BTA injection showed better outcomes with respect to the erythema index and skin elasticity (An et al., 2019). However, early BTA injections did not cause significant differences in the scar width and height.

**FIGURE 1:**
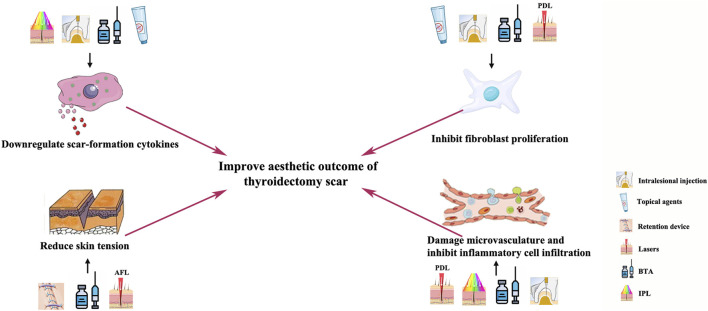
Mechanism of anti-scar methods for improving thyroidectomy scars.

Meanwhile, intralesional steroid injection has been widely used as a mainstay for scar management because it inhibits fibroblast growth and promotes collagen degeneration (Kelly, 2004; Zhang et al., 2016). Chemotherapy agents, such as 5-fluorouracil and bleomycin, which have been used in keloids (Nanda and Reddy, 2004; Tziotzios et al., 2012), could be carefully used in hypertrophic linear scars when satisfying results cannot be reached by a single steroid injection. The intralesional injection of steroids combined with 5-fluorouracil is suggested to be conducted at an early stage for patients with scarring predispositions (Srivastava et al., 2018). Therefore, when the scar appears to harden and swell after thyroidectomy, the above treatment methods can be used (Figure 1).

## Lasers and phototherapy

Various types of lasers and phototherapy methods, mainly including pulsed dye laser (PDL), intense pulsed light (IPL), ablative fractional laser (AFL), and non-ablative fractional laser (NAFL), have been used to improve the cosmetic outcome of the scar after thyroidectomy (Jung et al., 2011; Chung et al., 2021).

**FIGURE 2:**
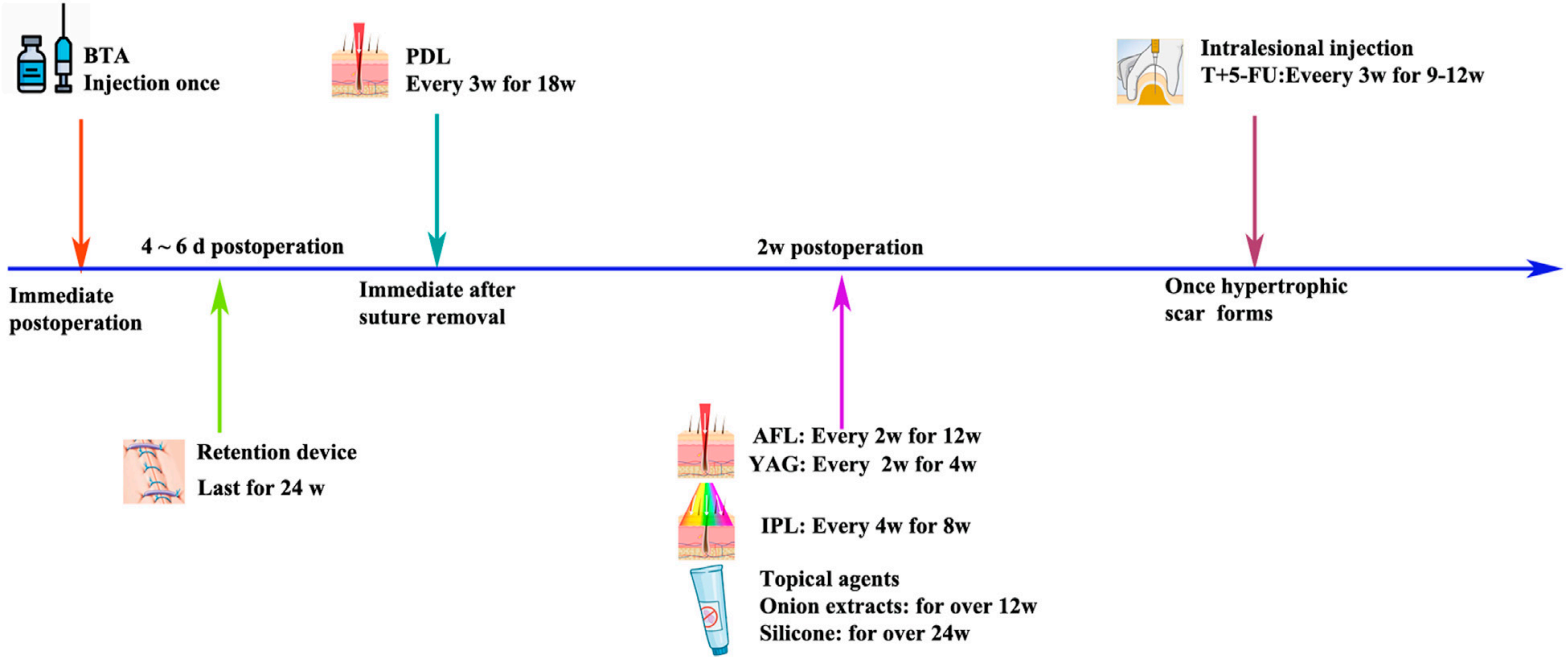
Intervention timing and treatment duration of thyroidectomy scar therapies.

IPL: IPL (400–1,200 nm; 500–600 nm) selectively targets hemoglobin in intravascular red blood cells to close local blood vessels and reduce blood supply for scar tissue growth (Fu et al., 2019). In a prospective study, the authors evaluated the safety and effectiveness of IPL in the treatment of burns, trauma, surgery, and acne scars with an initial treatment duration of 3 months and a treatment interval of 2–4 weeks, with patients receiving an average of eight treatments. IPL can not only effectively improve the appearance of hypertrophic scars and keloids but also reduce the height, redness, and hardness of scars (Erol et al., 2008). Some scholars also started IPL treatment immediately after removal, at 4 weeks and 8 weeks, and evaluated the therapeutic effect by using the Patient and Observer Scar Assessment Scale (POSAS) and Vancouver Scar Scale (VSS) scores, suggesting that IPL combined with erbium-doped yttrium aluminum garnet (Er:YAG) laser has a better preventive effect on scars than Er:YAG alone or no treatment (Kim et al., 2021). Therefore, IPL treatment of post-thyroidectomy scars should be carried out as early as possible, and effective treatment can be carried out within 6 months after surgery (Figure 2).

Pulsed dye laser, targeting the vasculature, has become a commonly used laser treatment for postoperative scarring. In 1993, the pulsed dye laser at 585 nm was reported to significantly improve erythematous and hypertrophic scars (Alster, 1993). In 2012, a randomized controlled trial with a follow-up time of 28 weeks proved that PDL treatment can improve the outcome of surgical scars in aspects of erythema, pigmentation, elasticity, and thickness (Davari et al., 2012). Although the exact anti-scarring mechanism remains unclear, previous literature proved that PDL emits light energy that is absorbed by hemoglobin, generating heat and resulting in damage to the microvasculature in the early scarring phase (Bouzari et al., 2007). Furthermore, 585-nm PDL is can decrease fibroblast proliferation and collagen type III deposition (Kuo et al., 2005). Several previous studies suggested PDL treatment should be conducted immediately after the removal of surgical stitches (Nouri et al., 2003; Alam et al., 2006; Conologue and Norwood, 2006; Nouri et al., 2009). However, for prevention of thyroidectomy scars, PDL treatment is suggested to be conducted 2–3 weeks after the removal of surgical stitches (Ha et al., 2014).

Ablative fractional laser based on the fractional approach, such as the 10,600-nm carbon dioxide (CO_2_) and 2,940-nm Er:YAG fractional laser system, is another common strategy for the early treatment of postoperative scars (Alster, 1999; Alster and Zaulyanov, 2007; Yun et al., 2011). AFL produces arrays of microscopic thermal wounds called microscopic treatment zones (MTZs) at specific depths. The intact epidermal architecture surrounding each MTZ rapidly heals, which in turn stimulates progressive collagen remodeling in scars (Manstein et al., 2004; Geronemus, 2006; Laubach et al., 2006). Several studies have proven the ablative CO_2_ fractional laser (CO_2_ AFL) system to be effective and safe in early postoperative interventions of thyroidectomy scars (Jung et al., 2011) and other surgical linear scars (Lee et al., 2013; Shin et al., 2014; Sobanko et al., 2015). Consensus has not been reached on the intervention time of CO_2_ AFL, with literature studies suggesting the time as on the day of suture removal (Sobanko et al., 2015), 2–3 weeks after stitch removal (Lee et al., 2013), and 2–3 months after surgery (Shin et al., 2014). However, one study suggested that 2–3 weeks after surgery could be an appropriate window for AFL treatment of thyroidectomy scars because re-epithelialization would be complete at this time point (Jung et al., 2011). The treatment energy of AFL should be carefully reduced to make the heat damage depth reach the dermis but without total penetration of the scar (Anderson et al., 2014) since another study found aggravation of hypertrophic scars when treating post-thyroidectomy scars 2–3 months after surgery, which is due to excessive AFL energy and density (Shin et al., 2014).

The development of non-ablative fractionated laser technology provided a new tool for the successful treatment of linear scars, offering the fractional thermolysis technique with minimal sequelae and a short downtime (Behroozan et al., 2006; Niwa et al., 2009). NAFL produces arrays of MTZs to stimulate collagen remodeling in scars, although sparing the epidermis while reaching a depth of 300–400 um in the dermis. Compared to NAFL, AFL caused more aggressive damage with the epidermis included in the MTZs, leading to slightly more side effects and a longer downtime, but ultimately has greater and more prolonged effects (Hantash et al., 2007a; Hantash et al., 2007b; Avram et al., 2009). Multiple studies have suggested that use of an erbium–glass (Er:Glass) NAFL for linear scar treatment might decrease the incidence of hypertrophic scarring and accelerate the improvement of postoperative scars (Choe et al., 2009; Ha et al., 2014; Karmisholt et al., 2018). The appropriate window for the NAFL treatment of the postoperative linear scar is unknown, with limited literature suggesting 2–3 weeks (Kim H. S. et al., 2012) or 2 months (Shin et al., 2014) after surgery. Of note, combination treatment, such as NAFL plus IPL (Kim et al., 2021) or NAFL combined with intralesional triamcinolone injection (Chung et al., 2021), achieves greater improvement in post-thyroidectomy scar prevention and management (Table 1).

**TABLE 1:** Treatment timing and duration of thyroidectomy anti-scar interventions.

	Intervention timing	Treatment duration	Invasive therapy
Topical agents			
Silicone products	2 w postoperation	24 w	N
Asiaticoside	2 w postoperation	12 w	N
Onion extracts	2 w after suture removal	>12 w	N
Skin tension-bearing device			
Skin load-bearing tape or device	4–6 d postoperation	24 w	N
Local injections			
BTA	Immediately after surgery	Once for 24 w	Y
Triamcinolone + 5-Fu	Hypertrophic scar formation	Every 3 w for 9–12 w	Y
Lasers and phototherapy			
Carbon dioxide AFL	2 w postoperation	Every 2 w for 12 w	Y
IPL	2 w postoperation	Every 2–4 w for 8 w	N
PDL	Immediately after suture removal	Every 3 w for 18 w	N
Nd:YAG	2 w postoperation	Every 2 w for 4 w	Y
NAFL	2 w postoperation	Every 4 w for 12 w	N

BTA, botulinum toxin type A; 5-FU, 5-fluorouracil; AFL, ablative fractional laser; IPL, intense pulsed light; PDL, pulsed dye laser; YAG, yttrium aluminum garnet; NAFL, non-ablative fractional laser.

## Conclusion

Early clinical management of thyroidectomy scars is a comprehensive procedure with a duration of 3–6 months. The most modifiable factor of scar prevention is the design of the skin incision and suture material, which leads to the least amount of wound tension in the postoperative period. Local injection of BTA, tension-bearing devices, and AFL might contribute to reduction in skin tension during the early post-thyroidectomy stage. In addition, local injection of steroids and chemotherapy agents, topical anti-scar agents, and PDL might contribute to the damage of the microvasculature and the decrease in fibroblast proliferation during the early thyroidectomy scar stage. A flexible combination and selection of these early postoperative interventions might help thyroidectomy patients achieve an optimal esthetic outcome.
